# Multilevel Predictors of Concurrent Opioid Use during Methadone Maintenance Treatment among Drug Users with HIV/AIDS

**DOI:** 10.1371/journal.pone.0051569

**Published:** 2012-12-12

**Authors:** Bach Xuan Tran, Arto Ohinmaa, Steve Mills, Anh Thuy Duong, Long Thanh Nguyen, Philip Jacobs, Stan Houston

**Affiliations:** 1 School of Public Health, University of Alberta, Edmonton, Alberta, Canada; 2 Institute for Preventive Medicine and Public Health, Hanoi Medical University, Hanoi, Vietnam; 3 Institute of Health Economics, Edmonton, Alberta, Canada; 4 Family Health International, Hanoi, Vietnam; 5 Administration of HIV/AIDS Control, Ministry of Health, Hanoi, Vietnam; 6 Faculty of Medicine and Dentistry, University of Alberta, Edmonton, Alberta, Canada; Yale School of Public Health, United States of America

## Abstract

**Background:**

Ongoing drug use during methadone maintenance treatment (MMT) negatively affects outcomes of HIV/AIDS care and treatment for drug users. This study assessed changes in opioid use, and longitudinal predictors of continued opioid use during MMT among HIV-positive drug users in Vietnam, with the aim of identifying changes that might enhance program efficacy.

**Methods:**

We analyze data of 370 HIV-positive drug users (mean age 29.5; 95.7% male) taking MMT at multi-sites. Opioid use was assessed at baseline, 3, 6, and 9 months using interviews and heroin confirmatory urine tests. A social ecological model was applied to explore multilevel predictors of continued opioid use, including individual, interpersonal, community and service influences. Generalized estimating equations (GEE) statistical models were constructed to adjust for intra-individual correlations.

**Results:**

Over 9 month follow-up, self-reported opioid use and positive heroin urine test substantially decreased to 14.6% and 14.4%. MMT helped improve referrals and access to health care and social services. However, utilization of social integration services was small. GEE models determined that participants who were older (Adjusted Odd Ratio - AOR = 0.97 for 1 year increase), had economic dependents (AOR = 0.33), or were referred to TB treatment (AOR = 0.53) were less likely to continue opioid use. Significant positive predictors of ongoing opioid use included frequency of opioid use prior to MMT, peer pressure, living with sexual partners, taking antiretroviral treatment, other health concerns and TB treatment.

**Conclusion:**

These findings show that MMT in the Vietnamese context can dramatically reduce opioid use, which is known to be associated with reduced antiretroviral (ART) adherence. Disease stage and drug interactions between antiretrovirals or TB drugs and MMT could explain some of the observed predictors of ongoing drug use; these findings could inform changes in MMT program design and implementation.

## Introduction

Scaling up antiretroviral treatment (ART) services in large injection-driven HIV epidemics has been challenged by multiple problems related to both drug use and HIV/AIDS [1]. HIV-positive drug users (DUs) usually delayed access and poorly adhered to ART, which are central to the success of HIV/AIDS treatment [1], [2]. Therefore, drug addiction treatment has become an essential component of HIV/AIDS Strategic Plans globally.

Medication-assisted treatment using methadone is one of the most efficacious treatments for opiate addiction [3]. Regular Methadone Maintenance Treatment (MMT) reduced the frequency of opioid use and risk behaviors, and improved ART adherence and quality of life outcomes in DUs with HIV/AIDS [4]. Effectiveness of MMT was consistent across culturally diverse settings, and between developed and developing countries [3], [4]. Nonetheless, low retention and drug abstinence rates remain a primary problem in treating addiction [5]. Substantial relapse and drop-out rates in MMT cohorts have been observed in various settings, due to multiple causes, including patients’ attributes, therapeutic process, environmental influences and program characteristics [6]–[13]. A better understanding of these barriers could guide program changes to improve retention and outcomes of MMT services [10].

The social ecological model is a helpful tool to recognize, explore and address multiple influences, which shape health behaviors [14]. The model describes influences of health behaviors at various levels, from individual, interpersonal, community to society [15]. These factors interact with each other to shape health behaviors of individuals. Notwithstanding, few researchers have applied this model in the study of ongoing opioid use during MMT, and little is known about injection-driven HIV epidemics in low-income settings.

**Table 1:** Baseline individual and familial characteristics of respondents and crude odd ratios for ongoing drug use in univariate GEE models.

Characteristics			Crude OR of Drug Use	
			(+) Urine Heroin Test	Reported Drug Use
**I. Individual**				
**1.1. Demographic**	**Mean**	**(95% CI)**		
Age	29.5	(28.9; 30.1)	0.98	0.98*
Income per month	5.78^b^	(5.16^b^; 6.40^b^)	1.06^a^	1.01^a^
	**N**	**(%)**		
Male sex	354	(95.7)	0.74	1.13
Ethnicity: *- Kinh people*	348	(94.0)	0.73	0.58
Religion*: - Buddhism*	351	(94.9)	0.74	0.39*
Under High school education	231	(62.6)	1.34*	0.97
Married	120	(32.4)	0.87	0.65**
**1.2. Morbidity**				
Taking ART	180	(48.7)	1.55**	1.27*
HBV	82	(22.4)	1.04	1.12
HCV	225	(61.5)	0.76	0.89
**1.3. History of drug use**	**Mean**	**(95% CI)**		
Age at first drug use	20.6	(20.1; 21.2)	0.99	0.99
IDU length	6.7	(6.3; 7.1)	1.02	0.98
Frequency of drug use (times/day)	3.2	(3.1; 3.4)	1.76***	n/a
	**N**	**(%)**		
History of drug detoxification	364	(98.4)	0.49*	0.59
Reason for drug relapsing:				
* Peers’ pressure and enticement*	212	(58.2)	1.71**	1.22*
* Desire, craving for drug*	247	(67.9)	1.14	1.55**
* Sad and disappointed*	164	(45.1)	1.12	0.92
History of overdose	75	(20.3)	1.13	1.06
**II. Family**				
Have economic dependents	10	(2.7)	1.30	0.40**
Cohabitants:				
* Parents*	316	(85.4)	0.70*	1.08
* Husband or wife*	100	(27.0)	0.81	0.63**
* Children*	99	(26.8)	0.94	0.59**
* Sibling*	213	(57.6)	0.88	1.19
* Relatives*	44	(11.9)	0.64*	1.07
* Partners*	13	(3.5)	1.86*	1.26
* Alone*	4	(1.1)	0.96	n/a

Note: * p<0.3; ** p<0.05; ***p<0.01.

a Odd ratio of higher income,

b Million Vietnamese dong in 2009 (1 USD = 17,800 dong).

n/a: not applicable.

Vietnam has a large injection-driven HIV epidemic with an estimated number of 254,000 people living with HIV/AIDS [16]. According to the National Committee for AIDS, Drugs and Prostitution Prevention and Control, 146,731 DUs have been recorded in 90% of districts in the country [17]. Although HIV prevalence among DUs moderately decreased over the last decade, from 29.4% in 2002 to 18.4% in 2009, DUs remained the largest at-risk group. Since 2008, the Vietnam Ministry of Health has been piloting the first national MMT program for DUs[18]–[20]. This service was introduced in Vietnam during a transition of HIV/AIDS care and treatment policies globally; with a call for earlier ART and more comprehensive interventions in drug using populations [2], [21]. Therefore, it was essential to determine how HIV-positive DUs respond to MMT, and which factors could influence the impact of MMT program on HIV/AIDS care and treatment in Vietnam. Moreover, our previous analysis showed that ongoing opioid use negatively affects health-related quality of life of HIV+ drug users[18], [22]–[24]. Consequently, understanding predictors of ongoing opioid use is necessary if one is to develop effective intervention packages for DUs and make programmatic decisions. In this analysis, we described changes in opioid use, and applied the social ecological model to identify longitudinal predictors of continued opioid use during MMT among HIV-positive DUs in Vietnam.

## Methods

### Ethics Statement

This study did a secondary analysis of data from a cohort study in Vietnam. The original project, which was conducted by the Vietnam Ministry of Health, was granted ethical approved from the Hanoi School of Public Health and the Ministry of Health. This secondary analysis, approved by the Vietnam Ministry of Health, was also granted ethical approval from the Hanoi School of Public Health, and from the University of Alberta’s Health Research Ethics Board.

### Study Design and Participants

We analyzed data from the pilot MMT cohort in Vietnam [18]. Two metropolitan areas with largely injection-driven HIV epidemics, Hai Phong and Ho Chi Minh City, were first selected for implementing the MMT program. They represent geographical areas with established HIV epidemics in large DUs populations, and settings where comprehensive HIV interventions have been implemented. Both Hai Phong and Ho Chi Minh City had interventions and projects supported by the National HIV/AIDS Program, The U.S. President's Emergency Plan for AIDS Relief (PEPFAR), the Global Fund to fight AIDS, TB and Malaria, the World Bank/DFID HIV project, and other global health initiatives. In 2009, Hai Phong had 31 harm reduction sites in 11 out of 14 districts targeting injecting drug users, sex workers and men who have sex with men; and 13 ART sites for adults, children and 9 antenatal care clinics for HIV-positive women. Meanwhile, Ho Chi Minh City had 80 harm reduction, 31 ART and 30 antenatal care sites, which were being operated in all 24 districts 29. Since 2009, the Vietnam Administration of HIV/AIDS Control has conducted a multi-site longitudinal cohort study of all DUs recruited from January 2009 to October 2009 at 3 MMT clinics in Ho Chi Minh City (District 4, District 6 and Binh Thanh District), and 3 clinics in Hai Phong City (Le Chan, Ngo Quyen, and Thuy Nguyen District). These clinics provided daily methadone for eligible DUs free-of-charge under direct observation of health workers. Patients at baseline were methadone-naïve, had a history of drug use, and did not have any severe health condition. All of them volunteered and gave informed consent to participate in the cohort study. Participants were tested for HIV at baseline. Repeated assessments of recruited DUs were conducted at baseline, 3 months, 6 months and 9 months. The majority of drug users in Vietanm (90%) were opioid dependent. Self-reported opioid use was obtained in interviews using structured questionnaires. Opioid (heroin) confirmation urine tests were done every 3 months. Of 968 patients recruited to the cohort, 370 (38.2%) were diagnosed with HIV at baseline and selected for further analysis.

**Figure 1:**
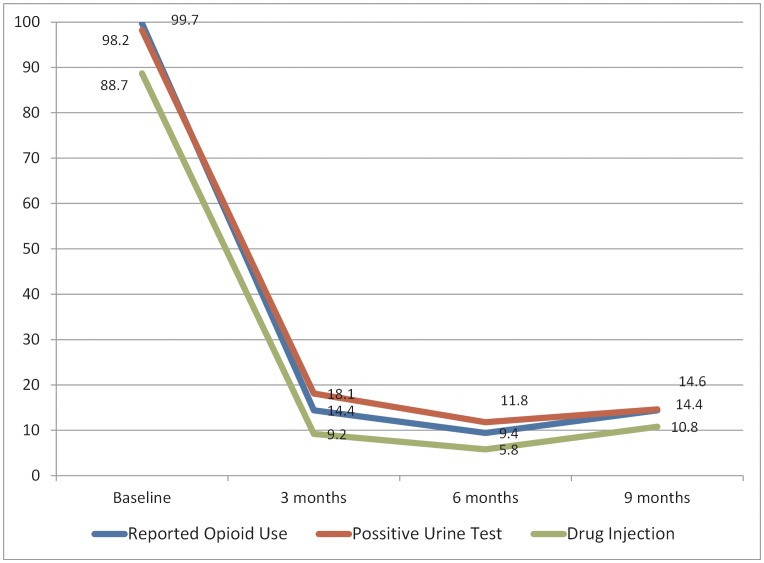
Percentage of ongoing drug use in HIV+ drug users over 9 month MMT cohort.

**Table 2:** Changes in individual, interpersonal and services-related predictors of ongoing drug use in univariate GEE models (N = 370).

Characteristics	Baseline		3 months		6 months		9 months		Crude OR of Drug Use	
	*n (%)*		*n (%)*		*n (%)*		*n (%)*		(+) Urine Heroin Test	Reported Drug Use
**I. Individual**										
**1.1. Demographic**										
Employment										
Unemployed:	147	(39.7)	142	(38.5)	128	(35.3)	107	(31.3)	1.00	1.20
* Unworkable due to illness*	64	(17.3)	32	(8.9)	29	(8.4)	19	(5.7)	1.27	1.21
* Can't get a job*	39	(10.5)	21	(5.9)	9	(2.6)	13	(3.9)	0.59*	1.11
**1.2. Morbidity**										
Had hospital admission last 3 months	131	(35.4)	9	(2.4)	12	(3.3)	15	(4.4)	1.20	1.12
Had health concerns	61	(16.5)	96	(26.8)	63	(18.2)	71	(21.1)	0.91	1.61**
**III. Interpersonal**										
Not satisfied with cohabitants	15	(4.1)	5	(1.4)	8	(2.3)	3	(0.9)	0.95	0.46
Have conflicts with others	79	(21.4)	24	(6.7)	24	(6.9)	16	(4.8)	1.14	1.32
Have IDU cohabitant	29	(7.8)	15	(4.2)	15	(4.3)	14	(4.2)	0.94	1.11
Had injected partners	45	(32.4)	15	(8.1)	18	(10.7)	21	(13.0)	0.98	1.00
**IV. Services access and utilization**										
**4.1. Referral**										
VCT	253	(68.8)	253	(70.7)	225	(64.8)	232	(69.1)	0.86	0.80*
OI treatment	106	(28.7)	140	(39.1)	149	(42.9)	161	(47.9)	0.72*	0.83
TB treatment	133	(36.0)	161	(45.0)	162	(46.7)	172	(51.2)	0.61**	0.64**
Mental health care	35	(9.5)	82	(22.9)	81	(23.3)	105	(31.3)	0.61**	0.59**
Other health service	32	(8.7)	28	(7.8)	13	(3.8)	15	(4.5)	1.32	1.29
Social integration services	71	(19.2)	93	(26.1)	125	(36.0)	135	(40.2)	0.74*	0.90
Peer-education	79	(21.4)	122	(34.1)	128	(36.9)	132	(39.3)	0.64**	0.73*
Social services(loan, housing,)	20	(5.4)	40	(11.2)	49	(14.1)	60	(17.9)	0.48**	0.40***
Legal services	10	(2.7)	27	(7.5)	33	(9.5)	46	(13.7)	0.48**	0.47**
**4.2. Utilization**										
VCT	222	(60.2)	220	(61.5)	119	(34.4)	131	(39.0)	1.01	1.19
OI treatment	20	(5.4)	16	(4.5)	18	(5.2)	44	(13.1)	0.97	1.19
TB treatment	26	(7.1)	12	(3.4)	10	(2.9)	24	(7.1)	1.31	2.36**
Mental health care	0	(0.0)	1	(0.3)	2	(0.6)	0	(0.0)	n/a	n/a
Other health service	15	(4.1)	12	(3.4)	6	(1.7)	10	(3.0)	0.90	1.21
Social integration services	13	(3.5)	21	(5.9)	22	(6.4)	17	(5.1)	1.08	1.37
Peer-education	28	(7.6)	33	(9.2)	25	(7.2)	27	(8.0)	0.50*	0.58*
Social services(loan, housing,.)	3	(0.8)	3	(0.8)	5	(1.5)	5	(1.5)	0.49	n/a
Legal services	1	(0.3)	3	(0.8)	0	(0.0)	3	(0.9)	n/a	1.29

Note: * p<0.3; ** p<0.05; ***p<0.01.

n/a: not applicable.

### Instruments and Measures

Opioid use, including heroin and other opiates, was self-reported by patients. In addition, heroin confirmation urine tests were conducted every 3 months. The primary outcomes of interest were continued opioid use in repeated assessments. This could be determined by either self-reported opioid use in the last month or positive heroin confirmation urine test at clinic visits.

The themes of the interview covered various levels of determinants influencing drug use behaviors. We used the social ecological model as the framework to explain predictors of ongoing opioid use during MMT. This model classifies influential factors into 4 fundamental levels: individual, interpersonal, community and society. The idea of this model emphasizes on continuous interactions between individuals with their physical and social environment that helps understand health behaviors as well as develop comprehensive health promotion interventions [14], [25].

Individual level included biological and personal history factors that may increase the likelihood of ongoing drug use. Socio-economic characteristics included age, gender, marital status, ethnicity, religion, education level, income, employment and ability to work. Morbidity included Hepatitis B and C Virus (HBV and HCV) co-infections, opportunistic infections, including TB, antiretroviral treatment, and other significant health concerns. History of opioid use included length of opioid dependence, age at the first opioid use, and frequency of use, overdose, drug detoxification and reasons of continued drug use prior to MMT.
Interpersonal level included influences of family members, peers, and intimate partners and their drug use behaviors on individuals.
Community and societal factors were those related to settings, infrastructure and broader social policies. In this study, we were interested in health care access through referral system, and availability and utilization of health and community support services, such as loans, legal support, education supports.

### Statistical Analysis

Descriptive statistical analysis was used to describe health status, socio-demographics, drug use behaviors, access and utilization of health care and social services. Associations between potential explanatory variables and continued drug use were first explored by univariate regression. In longitudinal assessments, patients’ characteristics and responses (e.g. ART, tuberculosis (TB) treatment, health services ulization,.) were repeatedly collected, therefore, independence assumptions of parametric models were violated, and parameter estimates would be biased. To accommodate the correlated nature of data, we used generalized estimating equations (GEE) for binary outcomes with a logit link to estimate the population-average effects of covariates on the mean outcomes [18].

**Table 3:** Multivariate GEE analysis of predictors for ongoing drug use during 9 month MMT in HIV+ drug users.

	Reported Drug Use			(+) Urine Heroin Test		
	AOR (95% CI)		p	AOR (95% CI)		p
**I. Individual**						
**1.1. Demographic**						
Age	**0.97**	(**0.94**; **1.00**)	**0.06**			
Religion: *- Buddhism*	0.41	(0.13; 1.28)	0.13			
Under High school education				1.30	(0.84;2.02)	0.24
Married	0.88	(0.36; 2.16)	0.78			
Employment:						
* Can't get a job*				0.46	(0.19; 1.13)	0.09
**1.2. Morbidity**						
Taking ART	**1.43**	(**0.96**; **2.13**)	**0.08**	**2.05**	(**1.36**; **3.09**)	**<0.001**
Had health concerns	**1.54**	(**1.01**; **2.37**)	**0.05**			
**1.3. History of drug use**						
Frequency of drug use (times/day)				**1.62**	(**1.18**; **2.23**)	**<0.001**
History of drug detoxification						
Reason for drug relapsing:						
* Induced by peers*	1.32	(0.89; 1.98)	0.17	**1.79**	(**1.16**; **2.74**)	**0.01**
* Desire, craving for drug*	**1.53**	(**0.99**; **2.37**)	**0.06**			
**II. Family**						
Have economic dependents	**0.33**	(**0.12**; **0.88**)	**0.03**			
Cohabitants:						
* Parents*				0.84	(0.53; 1.34)	0.46
* Husband or wife*	0.86	(0.33; 2.22)	0.76			
* Children*	0.81	(0.41; 1.62)	0.56			
* Relatives*				0.65	(0.32; 1.33)	0.24
* Partners*				**2.00**	(**0.94**; **4.25**)	**0.07**
**III. Interpersonal**						
Had injected partners	4.93	(0.66; 36.79)	0.12	2.94	(0.59; 14.59)	0.19
**IV. Services access and utilization**						
**4.1. Referral**						
VCT	0.97	(0.62; 1.52)	0.89			
OI treatment				1.29	(0.69; 2.44)	0.43
TB treatment	**0.53**	(**0.31**; **0.91**)	**0.02**	**0.55**	(**0.30**; **1.00**)	**0.05**
Mental health care	0.91	(0.50; 1.65)	0.75	0.80	(0.45; 1.42)	0.45
Social integration services				0.99	(0.61; 1.62)	0.98
Peer-education	1.19	(0.71; 2.01)	0.51	0.77	(0.44; 1.34)	0.35
Social services (loan, housing)	0.59	(0.25; 1.43)	0.25	0.77	(0.35; 1.70)	0.51
Legal services	0.78	(0.29; 2.10)	0.63	0.77	(0.32; 1.84)	0.55
**4.2. Utilization**						
TB treatment	**3.11**	(**1.46**; **6.60**)	**<0.001**			
Peer-education	0.62	(0.26; 1.50)	0.29	0.61	(0.28; 1.35)	0.22

Since estimation of parameters in GEE is performed on quasi-likelihood, standard model selection such as stepwise techniques and the Akaiki Information Criteria, which are based on likelihood methods, were not applicable [26]. Hence, in multivariate analysis, we applied the Quasi-likelihood Information Criteria to select “autoregressive” and “exchangeable” data working structures for GEE models of having positive heroin confirmation urine test and self-reported drug use, respectively [26].

## Results

### Socio-demographic Characteristics of Participants

After 9 month follow-up, 342 patients were retained (92.4% of baseline), of whom 337 (89.9%) received all 4 repeated assessments. Percentages of HBV and HCV positive individuals were 22.4% and 61.5%, respectively. With their first opioid use at a mean age of 20.6 (95% CI = 20.1; 21.2), participants had used drugs for an average period of 6.7 years (95% CI = 6.3; 7.1) prior to entering the MMT program (Table 1). Frequency of use was 3.2 times per day prior to MMT, and 20.3% had experienced an overdose. Heroin was the most common type of drug (95.1%), and 85.2% of respondents had a history of drug injection (data not shown). Almost all participants had been through some forms of structured detox programs (98.4%), such as compulsory drug rehabilitation centers for compulsory drug rehabilitation, and inpatient- or outpatient- detoxification; however, they reported relapsing because of craving for opioid (67.9%), feeling sadness and disappointment (45.1%), and peer influences (58.2%) (Table 1).

### Changes in Drug use Behaviors during MMT

The proportion of self-reported drug use last month and positive urine tests was initially 99.7% and 98.2%. During MMT, these indicators rapidly decreased to 18.1% and 14.4% after 1 trimester, and then plateaued over the next 2 trimesters. The kappa statistic of self-reported drug use and heroin confirmation urine test was 0.71 (86.7% agreement). Percentage of reported drug injection also changed accordingly, from 88.7% to 10.8% after 9 months (Figure 1).

### Changes in Access to and Utilization of Health and Social Services

Table 2 describes the changes in selected characteristics of respondents over 9 month MMT cohort. We observed a large reduction in the number of hospital admissions among respondents, but only a small change in the proportion having health concerns. A high proportion of participants had been referred over 9 months to health services, such as: voluntary HIV testing and counseling (VCT) (69.1%), treatment of opportunistic infections (OI) (47.9%), and TB treatment (51.2%). However, referrals to social support services such as social integration (40.2%), loan, housing (17.9%) or legal services (13.7%) were small. Noticeably, during the 3^rd^ trimester only 5.1% participants utilized social integration services, including vocational and skills training, and job referrals and placement services.

### Factors Associated with Ongoing Drug use during MMT in HIV-positive DUs

Table 1 and 2 present crude odd ratios of the associations between continued drug use and potential predictors in univariate GEE analysis. Having a positive urine test was significantly positively associated with: taking ART (OR = 1.55), higher frequency of drug use in the past (OR = 1.76 for increased 1 time/day), peer pressure and enticement (OR = 1.71), whereas negatively associated with: being referred to TB treatment (OR = 0.61), OI treatment (OR = 0.72), mental health care (OR = 0.61), peer-education (OR = 0.64), social services (OR = 0.48), and legal services (OR = 0.48). Significant positive predictors of reported ongoing drug use included: taking ART (OR = 1.27), peer pressure (OR = 1.22), craving (OR = 1.55), having health concerns (OR = 1.61), and receiving TB treatment (OR = 2.36). Besides referrals to health care and social services, protective factors of reported drug use also included marriage (OR = 0.65), living with wife or husband (OR = 0.63) and children (OR = 0.59), and having economic dependent (OR = 0.40).

Table 3 shows the adjusted odds ratios (AOR) in GEE models with multilevel predictors, including individuals, interpersonal, family, health care, and social services. Participants who had economic dependents (AOR = 0.33), or been referred to TB clinics (AOR = 0.53) were less likely to report ongoing drug use over 9 month MMT. Significant positive predictors of reporting continued drug use were: having health concerns (AOR = 1.54), and receiving TB treatment (AOR = 3.11). Craving (OR = 1.54) and taking ART (AOR = 1.43) positively predict drug use, however, this association had “borderline” statistical significance with p-values = 0.06 and 0.08, respectively. As for having positive urine tests, its significant predictors included: taking ART (AOR = 2.05), frequency of drug use prior to MMT (AOR = 1.62 for increased 1 time/day), and induced by peers (AOR = 1.79).

## Discussion

This study determined the changes in drug use patterns and multilevel predictors of continued drug use during MMT in HIV-positive DUs. We observed a high response rate (89.9%) and substantial reduction in drug use over 9 month MMT. We identified multilevel predictors of ongoing drug use during MMT, including morbidity (taking ART, TB treatment, had health concerns), pre- MMT drug use behaviors (frequency of use and craving), interpersonal and familial attributes (peer pressure and cohabitants), and access and utilization of health care and social services.

Individual characteristics positively associated with ongoing opioid use, such as higher historical frequency of opioid use and relapse prior to MMT, are consistent with findings of other studies in drug treatment [4], [7], [11], [27]–[29]. In addition, significant predictors emerged from this analysis were interpersonal and social interactions at family, community and service delivery levels. We observed protective factors to drug use, including marriage, living with wife or husband and children, access to health care, peer education, social integration and legal services. Meanwhile, factors with strong positive association with continued drug use were: peer pressure and enticement and living with sexual partners. Those patients, who have unstable family and social status, showed greater likelihood of continued drug use. This finding underlines the importance of the social context and drug network to the provision and success of addiction treatment [15].

In Vietnam, the HIV epidemic is concentrated on high-risk groups including injecting DUs and commercial sex workers, and a large proportion of sex workers are also using drugs. These high-risk behaviors are illegal and characterized as “social evils” in Vietnamese society. Thus, there might be hidden networking to facilitate illicit drug use. This can lead to group pressure on MMT patients to continue their drug behaviors if they do not develop a more stable social status. The impact of MMT programs, therefore, could be limited if other social supports services were not in place [18], [30], [31]. It is important to enable patients to adopt new healthy behaviors, and improve their social functioning by integrating them into the workforce and education system [32]. Vocational training and livelihood support services for those who are taking MMT may also be interventions that provide long-term achievements.

The large proportion of respondents with self-reported health concerns and co-morbidities indicated higher health care needs of HIV-positive DUs than HIV-negative ones. Poor health status in this population was indicated both by reported health concerns, and by antiretroviral therapy since official policy during the study period recommended ART initiation only for patients whose absolute CD4 cell count was <250 cells/ml. The causal relationship between drug use and deterioration in health status could be bidirectional – drug use would be expected to lead to more severe deterioration in health status. It is also possible that advanced disease stage could increase the risk for continued drug use, which would explain the observed association between continued drug use and ART use. During the period of this study, the national ART guidelines determining the first-line regimens included Zidovudine/Stavudine+Lamivudine+Nevirapine for main regimens, and Zidovudine/Stavudine+Lamivudine+Efavirenz for alternative regimens (Ministry of Health 2009). The second-line regimens are available for patients who had virological failure accounting for about 3% of the total ART patients. In the literature, certain interactions have been observed between methadone and antiretroviral medications in drug users with HIV/AIDS [33]. Our analysis showed a greater likelihood of continued drug use in patients taking ART or having TB treatment. Both NNRTI antiretroviral drugs and even more, rifampin, lower serum methadone levels through induction of hepatic sector, enzymes. As for TB patients, service referral was a protective factor, but TB treatment was a significant predictor of ongoing drug use. The contradictory associations indicate that the disease doesn’t contribute to ongoing drug use, but the treatment of TB might increase its likelihood. These effects could be an important part of the explanation for the observed association between ART use or TB treatment on one hand and continued opioid use. The program training and implementation should systematically incorporate awareness of potential drug interactions and anticipate the need for methadone dose adjustment in patients on ART or TB therapy.

The strength of this study was the longitudinal assessments of a sufficient sample that supports the causal inference of multilevel predictors of ongoing drug use. In addition, this study utilized the social and ecological model as a theoretical framework for identifying multilevel factors associated with the effectiveness of MMT programs in drug users with HIV/AIDS. Future evaluation studies could use the same approach to widen our understanding of effective treatments for opioid dependence in this population. However, several limitations of this study should be acknowledged. Given the limitation of a secondary analysis, we could not incorporate some important predictors at the interpersonal and community level. Therefore, the social ecological model was applied as an approach for classifying and identifying factors at different levels that were related to and could inform the improvement of MMT program once it’s expanded. As for drug use behaviors, patients recalled and self-reported that might lead to information biases. However, previous studies showed that reported drug use of those recruited through a community-based or out-reach program to be both accurate and reliable, and these findings were confirmed in our study by the good correlation with objective urine opiate testing [34]–[36]. Secondly, the 9 month follow-up might not be sufficient to assess the sustainability of this intervention. Besides, we did not have information on severity of ongoing drug use or methadone dosages given the limitation of secondary data use. Finally, in this analysis, we did not have a comparison group of those who were HIV-positive DUs without MMT, and the selection of MMT was on a voluntary basis that limits the casual inference and generalizability of the findings. Notwithstanding, as the first national pilot MMT program, the results of this study are useful for developing comprehensive HIV/AIDS care and treatment for HIV-positive DUs in Vietnam, as well as in other injection-driven HIV epidemics. This experience could prove useful to other programs in resource limited settings.
